# PathGame: Crowdsourcing Time-Constrained Human Solutions for the Travelling Salesperson Problem

**DOI:** 10.1155/2019/2351591

**Published:** 2019-05-13

**Authors:** Slaviša Dumnić, Đorđije Dupljanin, Vladimir Božović, Dubravko Ćulibrk

**Affiliations:** ^1^Department of Traffic Engineering, Faculty of Technical Sciences, University of Novi Sad, Trg Dositeja Obradovica 6, 21000 Novi Sad, Serbia; ^2^Faculty of Mathematics and Natural Sciences, University of Podgorica, Bulevar Džordža Vašingtona bb, 81000 Podgorica, Montenegro; ^3^Department of Industrial Engineering and Management, Faculty of Technical Sciences, University of Novi Sad, Trg Dositeja Obradovica 6, 21000 Novi Sad, Serbia

## Abstract

Human strategies for solving the travelling salesperson problem (TSP) continue to draw the attention of the researcher community, both to further understanding of human decision-making and inspiration for the design of automated solvers. Online games represent an efficient way of collecting large amounts of human solutions to the TSP, and PathGame is a game focusing on non-Euclideanclosed-form TSP. To capture the instinctive decision-making process of the users, PathGame requires users to solve the problem as quickly as possible, while still favouring more efficient tours. In the initial study presented here, we have used PathGame to collect a dataset of over 16,000 tours, containing over 22,000,000 destinations. Our analysis of the data revealed new insights related to ways in which humans solve TSP and the time it takes them when forced to solve TSPs of large complexity quickly.

## 1. Introduction

Finding the shortest path to visit several destinations is one of the problems humans have evolved to solve on a daily basis. In the scientific literature, this problem is known as the TSP, as it can be thought of as a problem of finding ways in which one may find the shortest path for a “salesperson,” who needs to visit a predefined set of “cities”. The salesman is required to visit a city only once and, in the closed-form variant of the problem, to return to the starting node.

Since there are a number of applications in the domain of transport and delivery, which would benefit from ways to determine an optimal path through a set of points, the earliest published literature on the TSP dates to the 18th century [1], although it has probably been studied much earlier than that. Perhaps surprisingly, the research has revealed that it is a complex problem and one that belongs to a larger group of problems for which no algorithm that can solve them in polynomial time is known at this point. TSP is perhaps the best-studied example of a nondeterministic polynomial time (NP) problem and is known as NP-complete [1]. It is worth noting that there is also no theoretical proof that a good algorithm for solving this class of problems does not exist. Thus, we do not really know how hard of a problem TSP is, except through empirical evidence that we have been unable to solve it adequately for quite some time; despite the large interest, it continues to draw from the scientific community.

Despite the fact that computers are unable to solve the problem in polynomial time, humans are able to do so for relatively small instances of the problem (10–120 points), in time that is a close-to-linear function of the problem complexity [2, 3].

When solving the TSP, humans rely more on their perceptual skills than cognitive skills. In an early (1974) study aimed at examining this aspect of the problem, Polivanova compared human performance on geometrically represented instances of the problem versus performance on instances where the travel costs were given explicitly for each pair of cities. The participants showed significantly better results on geometric instances [4].

It is no surprise that there have been attempts to create computational models that would match human performance on TSP.

Best and Simon [5] showed early on 2000 that 80% of all subject choices as to what node to go to next can be explained by following the nearest neighbor path.

At about the same time, MacGregor et al. [6] created a model which starts from the convex hull of the destination points and iteratively refines it to include all the points. Since the refinement procedure starts from a random point on the convex hull and they consider two different point inclusion criteria, their model provides a number of solutions for a given set of points. The model was evaluated on a range of data gathered from humans in their and previous studies dealing with 10, 20, 40, and 60 node problems, a single 48-node problem, and a single 100-node problem. The data used to evaluate the model varied in terms of the number of instances and subjects that generated it. For the 10–60 node problems, 50 undergraduate students generated the data, for “several” random problems with each number of nodes. For the 48-node problem, 103 human solutions were gathered, while for the 100-node problem, just 8 subjects solved the problem. Although it was tested on limited data available, the model proposed by MacGregor et al. matches human performance well.

In the time following MacGregor et al.'s study, there has been a number of papers aimed at studying a different aspect of human TSP solving, often proposing and refining human-inspired computational models for TSP solving. In their 2011 paper, MacGregor and Chu provide a good overview of more recent results [7]. We limit our discussion to the studies closely related to our work.

Human performance on Euclidean and non-Euclidean TSPs was examined by Walwyn and Navarro [8]. Their experiments, conducted on 40 participants (mostly undergraduate students), each of whom solved 12 10-point and 6 40-point problems on paper, support the hypothesis that humans create near-optimal solutions for both types of the problem while being slightly better at solving the Euclidean version.

Despite the perhaps obvious benefits that might be gained through the uses of modern information technology and video games in terms of gathering much larger sets of experimental data, there have not been many such approaches reported in the literature. In fact, the only study taking this route that we could identify is reported on in a 2017 paper by Rach and Kirsch, who created an online game Perlentaucher [9]. The main goals of their study were to address a large number of participants, to collect data on different problem variants, to create a game that does not feel like a test and to collect data on human solution procedures when provided with tools, and to identify the ability to repeat a task. The first three goals dealing with gathering data from a large number of participants, data for different problem variants and creating a game that does not feel like a test, coincide with the goals of the study presented here. However, in the present study, we are interested in larger non-Euclidean problems, so our design choices have been made accordingly.

If one wants to collect large amounts of data, an online game, interesting enough for the participants is a good solution. The study conducted by Rach and Kirsch showed that the solutions collected through online gameplay corresponds well to the data previously reported in the literature and the validity of such an approach to data collection.

**Figure 1:**
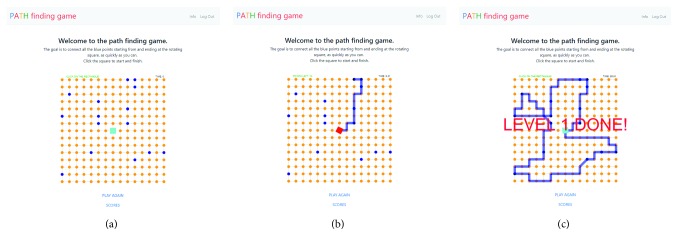
PathGame. (a) The random generated points. (b) The segment of the tour. (c) Completed tour.

The work of Rach and Kirsch is the one most related to the work presented in this paper (PathGame). Both approaches are based on an online game that can be played on devices running a modern browser and required a mouse or touchpad as an input device. PathGame support for touchscreen devices is being developed but is still not available at the time of writing. However, significant differences exist between the two approaches. The study of Rach and Kirsch relied on 24 instances of Euclidean TSP as stimuli. In PathGame, the number of points ranges from 10 to 196 and is stochastically determined, by specifying only the probability of a point being a destination point. The playing field, shown in Figure 1, consists of a rectangular grid of 15 × 15 (225) points and the score is calculated based on Manhattan (block-based), not Euclidean distance. To induce the players to follow non-Euclidean-based strategies, the game plots the resulting block-based path between the last destination point visited and the current position of the mouse pointer.

Formulating the TSP in terms of a sequential decision-making game has implications far beyond simple data gathering and human problem-solving faculties analysis. In the last few years, advancements in the field of AI have led computers to achieve beyond human performance in domains such as classic video games [10] and even the game of Go [11]. While this can be done without human supervision, human-generated data can be used to speed up the process significantly and, therefore, address more complex problems [12].

The study presented here is the first step towards creating an environment which can be used to train an AI agent that would solve non-Euclidean TSPs of any size efficiently. To this end, we have created an online game (PathGame) to collect human non-Euclidean TSP solution data, which can both be used to aid the training of such an agent and to further our understanding of human performance on this problem. Both the source code of our solution and the dataset collected during this trial study is available to researchers for free use in future studies (http://dculibrk.github.io/pathgame).

## 2. Experiment and Methods

### 2.1. Game Design

The design choices in our work were inspired by the primary goals of the study, which were formulated as follows:

1. Create a game that would enable collecting large amounts of human solutions to the non-Euclideanclosed-form TSP
2. Collect data that would enable subsequent training of a human-inspired AI agent focused on the same task

The focus on the closed-form non-Euclidean variant of the TSP is due to practical considerations. The intended prospective application of our AI agent is the optimization of ground transport (delivery) problems in an urban scenario. Thus, the agent should find the optimal route to make a number of deliveries in an urban environment, starting from a predefined depot and returning to the depot once the job is done. The city-block or Manhattan distance is a better approximation of the constraints of a ground vehicle moving through an urban environment than the Euclidean.

To induce “non-Euclidean” thinking in the players and represent the space graphically, we created an online game with a fixed playing field consisting of a rectangular grid of *N* × *N* points (as shown in Figure 1(a)). The dimension *N* of the grid is a parameter in the code of our game and can be easily modified. Within this fixed grid of points, the destination points are coloured blue, while the rest are orange. The starting point (the depot) is in the middle of the playing field and is indicated by the cyan rotating rectangle. The rectangle needs to be clicked to start the game. Once clicked, the rectangle changes colour (becomes red) and the text just above the playing field, instructing the user to “Click the rectangle to start!,” is replaced by a counter showing the number of destination points left for the user to visit and the time elapsed since the start of this particular session of the game. Destination points are visited, i.e., added to the path, when the mouse pointer is dragged over them. When this happens they turn black.

To further enhance the nonEuclidean character of the gameplay, we introduced a snap-to-grid behaviour of the path. As the user moves the mouse during gameplay, a potential path segment is generated from the last destination visited (or the depot, if the user just started) to the mouse pointer. The segment will be added to the path only when the user drives the mouse pointer over a new destination point. Thus the path is constructed segment by segment until all the destination points are visited. If instead of visiting a destination point, the user moves back towards the start of the potential path segment, the segment will be shortened, allowing the user to change the route. This is illustrated in Figure 1(b).

When all of the destination points have been visited by the user, the destination point counter is replaced by the text “Click the rectangle!,” instructing the user to visit the depot. When the user clicks the depot, the text “LEVEL DONE!” is flashed over the playing field, as shown in Figure 1(c).

In our design, we opted for a simple solution where each level corresponds to a single game played.

For the training of our AI agent, we would like to capture the instinctive actions of the players; rather than those taken after a long period of deliberation, we (just above the playing field) instruct our players that “The goal is to connect all the blue points starting from and ending at the rotating square, as quickly as you can.”

The destination points are determined in a uniformly random way before the start of each level, based on the probability controlled by the level (the number of games that the user played):

(1) $$\begin{array}{c} \text{prob}=\left\{\begin{array}{cc} \alpha *\text{level}+{\text{prob}}_{0}, & \text{level}\leq \theta ,\\ \alpha *\theta +\beta *\left(\text{level}-\theta \right)+{\text{prob}}_{0}, & \text{level}>\theta , \end{array}\right. \end{array}$$

where the prob (equation (1)) is the probability of each point in the grid being a destination point, *α* is the increase in the probability that is gained by completing each level before reaching a predefined threshold *θ*, *β* is the increase gained for each level after that, and prob_0_ is the initial probability of a destination point. The use of the piecewise linear function for prob was motivated by the desire to get a larger diversity of data, both in terms of the number of users and destinations early on, but to then switch to a mode where the number of destinations increases more slowly. The latter both makes it harder for our playing field to saturate with destination points and makes it harder to increase the score of the users (equation (2)), keeping the game fun for the more persistent.

To motivate our players, we calculate a score (equation (2)) for each game, which depends both on how short the path is, as well as how fast one solves the problem. Therefore our score is calculated as

(2) $$\begin{array}{c} \text{score}=\left(C_{1}+C_{2}*D-\frac{l\;\;}{\;\;D}-t\right), \end{array}$$

where *C*_1_ and *C*_2_ are constants designed to keep our score positive, *D* is the number of destination points in the problem, *l* is the length of the solution path, and *t* is the time it took the user to solve the problem. The shorter the length of the path, scaled by the number of points and the shorter the time it took to solve the problem, the higher the score. Path length (*l*) and time (*t*) are measured in pixels and milliseconds, respectively.

### 2.2. Implementation

Pathgame has been implemented in Python and JavaScript using Paper.js and Flask. Paper.js is an open source vector graphics scripting framework that runs on top of the HTML5 Canvas [13]. Flask is a web development microframework for Python [14].

### 2.3. Participants

The participants were mainly students and employees of the Faculty of Technical Sciences, University of Novi Sad, Serbia, and the Faculty of Mathematics and Natural Sciences, University of Podgorica, Montenegro. The students were mostly undergraduate students of Industrial Engineering and Management, Information Technology, Transport Engineering and Mathematics.

The participation was voluntary, and for the most players, not rewarded. A small reward in terms of course credit (corresponding to 5% of the full course credits) was provided for the students that were ranked among the top 10 players at a predefined time.

The participants were not required to divulge any personal details but were given an option to enter their age and sex. All chose to do so.

The total number of participants who participated in the experiment was 164, of which 59 (36%) were female and 101 male (64%). It is worth noting that when one takes into account only the top 10 players, they are perfectly balanced in terms of gender, as there are 5 women and 5 men there.

The oldest participant was 49 old and the youngest 19. 82% of the participants were aged between 19 and 24 years, 11% were between 25 and 29 years of age, while just 7% were 30 or older.

### 2.4. Procedure

We deployed our application on the Heroku cloud platform [15], which made it playable on any device with a modern web browser. Support for touchscreen devices has not been provided, as the size of the screens on these devices would need to be considered and the playing field scaled to enable comfortable playing. However, users were provided with a customized interface for mobile devices, which allowed them to check the high scores.

The link to the game was shared with the players through e-mail and the Faculty of Technical Science's e-learning platform. The web application required the users to register and login before they are able to play the game. A short explanation about the purpose of the research and the creators of the game was provided, and the users were required to give consent for the data collected to be used for research while preserving their anonymity at all times.

Once logged in, the users were free to play the game but were not able to replay the same level.

The deadline for reaching the top 10 rank in order to gain course credit was set to 10 days from the initial distribution of the link, and most of the data used in this study have been collected in this interval.

In our experiment, *θ* was set to 60 and prob_0_ to 0.01, while *α* and *β* were set to 0.1 and 0.01 initially. During the experiment, we changed the value of *β* twice, lowering it to 0.001 and then raising it to 0.005, to keep the game interesting for our players. The values used for *C*_1_ and *C*_2_ were 3000 and 10, respectively. *C*_2_ corresponds to the smallest number of destinations in our dataset. *C*_1_ was determined empirically, based on the initial testing, and corresponds to a value slightly above the absolute value generated by the other terms of equation (2) in the initial test runs.

## 3. Results and Discussion

**Figure 2:**
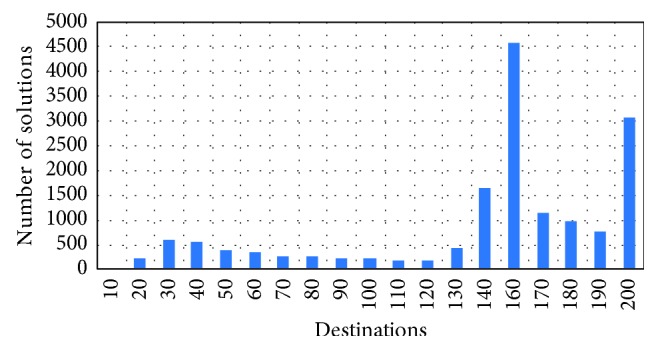
Distribution (histogram) of the solved TSP complexity.

A total of 16,053 TSPs were solved by our players. The distribution of the solutions in terms of a number of destination points is shown in Figure 2, and the solution complexity ranges from 10 destination points to 196. The maximum value actually corresponds to the probability of a point being a destination equal to 1, but we do not allow for destinations overlapping with the depot or along the row and column in which the depot finds itself. Effectively, this separates all the destination points in 4 quadrants. The largest number of solutions collected contained between 150 and 160 destination points. Our most frequent problem size is therefore significantly larger than what was considered in classic studies, which limit themselves to problems ranging to 120 points, and even more so when compared to other online game approach, Perlentucher [9], who considered problems of up to 20 destinations. In total, the tours we collected contain over 22.5 million destinations. Although Perlentucher study collected more tours (38,465), even if one considers that all their tours were of the largest problem side considered (20), the total number of destinations in that dataset is environed 770 thousand, making our dataset the largest in terms of total number of destinations considered.

For each tour, we calculated the total Manhattan distance from the depot, through all the points back to the depot, expressed simply in pixels, as we have a static size of the field (500 × 500 pixels).

Following the procedure used in [2], we compared our users' performance to the “optimal” solution determined using simulated annealing (SA) [16]. In addition, we compared their performance to the greedy nearest neighbor heuristic, which has both been showed to account for 80% of all user actions by Best and Simon [5] and is also the algorithm a naïve AI agent is likely to follow. For the SA experiments, we used the open source implementation available at [17], modifying it to support Manhattan distance. The default parameters for the SA were used, with the starting temperature set to 25,000 K and the ending to 2.5 K, while the number of steps was set to 100,000. Since the solutions produced by the SA vary from one run to the other, we ran the code 10 times for each problem in our dataset and initialized the SA with the NN solution, in an effort to produce solutions as close to optimal as possible. The best solution produced over the 10 runs was used in all analyses performed. SA took up to 10 seconds to produce the result per problem on our hardware.

**Figure 3:**
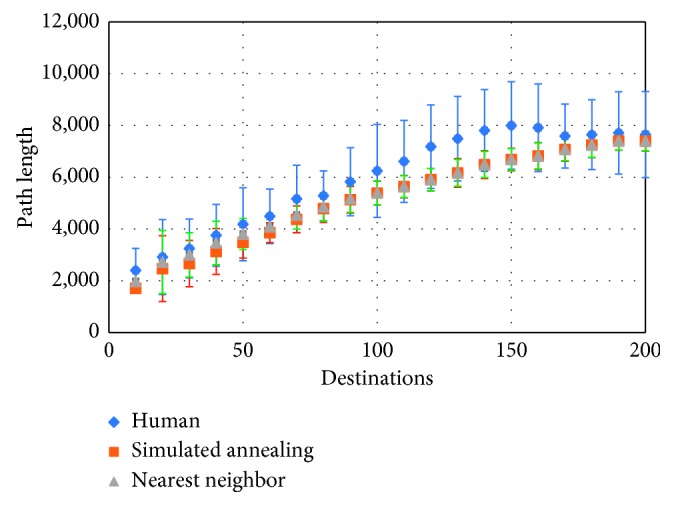
Distribution of solution lengths for different TSP sizes.

A box plot of the distribution of the path lengths achieved by the human participants, NN, and SA heuristic is shown in Figure 3. Binning into intervals, 10 nodes wide was used to produce the plot, similar to the approach used to generate Figure 2. As Figure 3 shows, humans, on average, do worse than both heuristics, and human-generated solutions exhibit the largest variance. The divergence between the human and machine-generated solutions increases with the complexity of problems but converges for problems larger than 150 destinations. The SA outperforms the NN heuristic for smaller problems, but the SA fails to improve on the NN solution for the larger problems.

ANOVA was used to confirm that there are indeed statistically significant (at the *p* < 0.05 level) differences in terms of path length between human and computer-generated solutions to the same problem. Overall the mean path length of human solutions is 7095 pixels, with a standard deviation of 2108 pixels, the nearest neighbor algorithm yields paths of the average length of 6350 pixels, with a standard deviation of 1394 pixels and the simulated annealing averages 6303-pixel paths, with the standard deviation of 1490.

**Figure 4:**
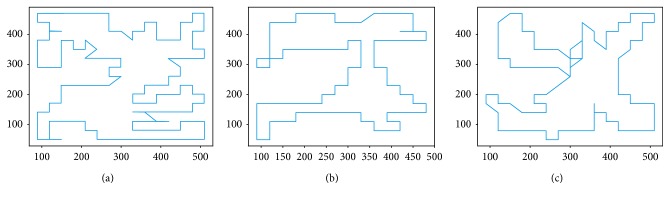
Sample tours where users beat the nearest neighbor.

Of particular interest to understanding, human TSP solving and creating human-inspired AI agents are the cases in which the humans do better than the heuristics. These cases comprise 26.72% of the whole dataset (4286 tours to be exact), which is slightly higher than the 80% rate reported by Best and Simon [5] as the percent of moves in which humans follow the nearest neighbor heuristic.  Figure 4 shows some such tours, generated by three different users. Although our human users sometimes used the ability to follow short direct paths, rather than adhering to the snap-to-grid policy, this did not affect the path length and the score, which are still calculated using Manhattan distance.

**Figure 5:**
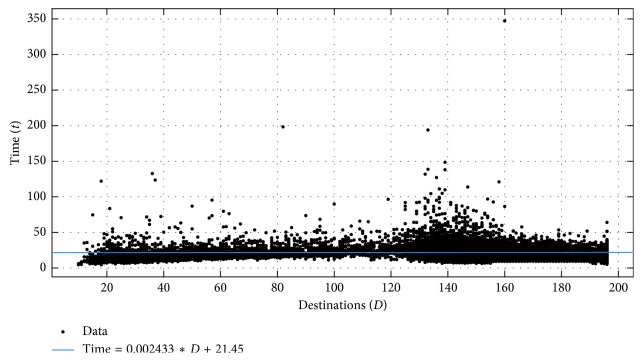
Time taken to solve the problems of different complexity.

In our study, we are interested in the tours people create when instructed to do so as quickly as possible, in order to capture the instinctive behaviour, rather than results of long deliberation. This had surprising consequences, as illustrated by the scatter plot in Figure 5.

Although the users in our study seem to solve the small problems of up to 30 points in time that linearly depends on the complexity of the problem, this trend did not continue for larger problems. In fact, users seem to reach a plateau in the time it takes them to create the tours after this point, and this plateau is maintained across all problem sizes. To check this statistically, a linear model was fit to the data using Matlab's curve fitting toolbox [18] and the linear least squares technique. The root-mean-square error (RMSE) obtained was 10.783, and the model had a negligible slope (*a* = 0.002443 with 95% confidence bounds (−0.0008836, 0.005769)). The model is indicated by the blue line in Figure 5. The intercept obtained (b) was 21.45 with 95% confidence bounds of (20.95, 21.95). The model indicates that our users seem to take around 21 seconds on average to solve the problem, regardless of the complexity and that this changes very little over time, which is probably due to the requirement to solve the problem as quickly as possible.

**Figure 6:**
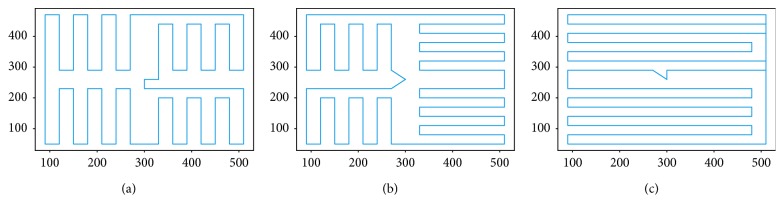
Sample solutions when destination points are numerous.

The plot in Figure 5 shows increased variance that corresponds to the two most populous bins in Figure 2, which is to be expected as most of our data correspond to problems of this size and probably does not indicate a trend of increased time needed to solve problems of this size. This is followed by the section in which the time taken to solve the problem actually decreases. While this initially seemed to be counterintuitive, closer inspection of the solutions reveals that, for the problems of this size, the distribution of destination points is so dense and that our users settled on solving the problem by using a predefined geometric pattern (route), rather than trying to optimize it. Some such solutions are illustrated in Figure 6.

**Figure 7:**
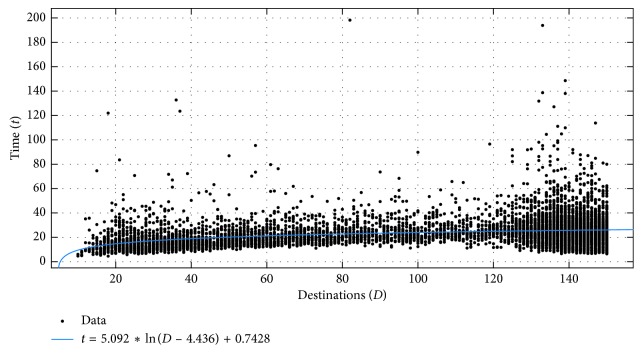
Time taken to solve the problems with less than 150 destinations.

This transition to geometric solutions seems to coincide with the inflection point that can be observed in the distribution of the human-generated solution length, shown in Figure 3. Since the nature of the data obtained after the inflection point is qualitatively different than those before it, we conducted a separate analysis for problems with 150 destinations or less. The distribution of the times taken by the users to solve the problem for these data, which contained 8146 samples, is shown in Figure 7. The analysis of the behaviour for the geometric solutions merits a separate study, which we plan to do in the near future.

Akin to the procedure followed by Dry et al. [2], we attempted to fit different functions to the relationship between the time and the number of destinations for the data in Figure 7, once again using Matlab and nonlinear least squares, except in the case of the linear model, where linear least squares was used. The results of this analysis are presented in Table 1.

RMSE and sum of squares due to error (SSE) were used to evaluate the goodness of fit. Since we do not have data for problems less than 10 points, we added a parameter that allows our models to intersect the *X* axis, as well as the *Y* axis. We do not report the Bayesian information criterion [2] in the Table 1 since the number of data points on which we fit our models is constant, and the only model that has 1 parameter less than the rest is the linear model, so the BIC corresponds to the SSE with a relatively small constant term added (∼27 in the case of the linear model and ∼18 in all other cases). Of all the models evaluated, the best is the first, which supports the hypothesis that there is a logarithmic relationship between the number of destinations and the time taken to solve the problem, in our specific experimental setup.

**Figure 8:**
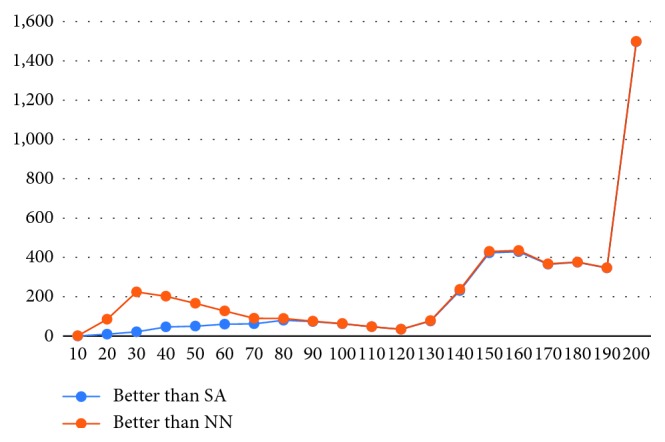
Distribution of solutions beating heuristics.

The cases in which users do better than the heuristics are of particular interest for AI applications. The distribution of human solutions outperforming the two algorithms evaluated is shown in Figure 8. For the problems of small size, the users rarely beat the computer, but the number rises significantly when the problem complexity reaches 150 destination points.

**Figure 9:**
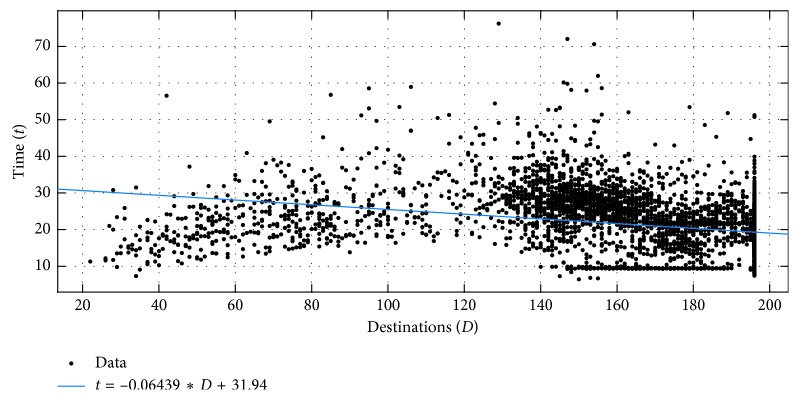
Time taken to create solutions better than those of the nearest neighbor and simulated annealing, plotted against the complexity of the problem.

When examining the time taken to create the solution in these cases (Figure 9), a pattern similar to that shown in Figure 7 can be observed. The users seem to take less time to solve progressively more complex problems, after a certain point. A linear regression fitted to the data, indicated by the blue line in Figure 9, shows this clearly, with a slope of −0.06439 and 95% confidence bounds of (−0.07058, −0.05821). Once again, this seems to be due to the “geometric pattern” routes, which are indicated by the horizontal line on the plot, as the users take very similar time to complete such routes (environ 10 seconds) and the routes are independent of the actual number and distribution of destination points. The plot also shows that the users start this behaviour when the number of destinations reaches about 150 points, which represents 67% of all the points in our grid. An AI agent might consider this in its problem-solving and default to following a predefined or learned geometric pattern to complete the tour in such cases.

**Figure 10:**
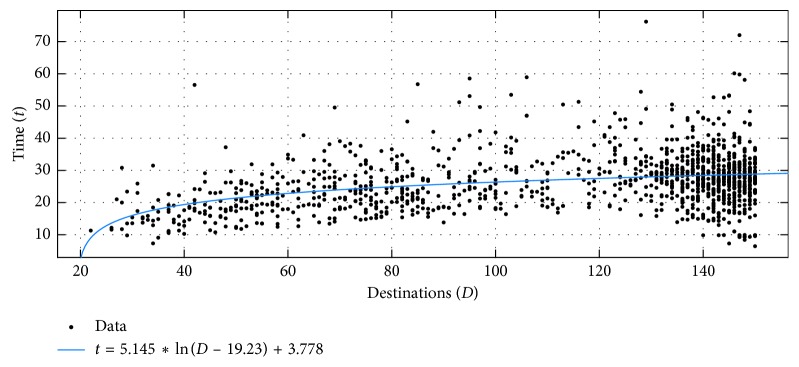
Time taken to create solutions better than those of the NN and SA, plotted against the complexity of the problem (for problems of 150 destinations or less).

When considering the solutions surpassing the two heuristics used, but limited to problems with 150 points or less (Figure 10), so that the geometric solutions are filtered out, the distribution is best described by a logarithmic function. We followed the same fitting procedure that was used to generate data in Table 1 and evaluated all the models. The logarithmic was the best model (RMSE 7.9286, SSE 77,383), followed by the linear (RMSE 8.0194, SSE 79,232). The best model is shown as a blue line in Figure 10. The 95% confidence bounds for the best model's coefficients are *a* (3.752, 6.538), *b* (2.988, 10.54), and *c* (−28.06, −10.4).

Finally, we have tried to determine whether the users benefit from their experience when it comes to the time it takes them to solve the problem. To do so, we examined the correlation between the average time a user takes per destination point and the number of games she/he has played. There is a moderate negative correlation between the two variables (−0.421, significant at the 0.01 level), suggesting that the users indeed seem to take less time per point as they gain experience but that the effect is not that pronounced.

## 4. Concluding Remarks

We have presented a web-based gaming solution (PathGame) designed to enable the crowdsourcing of human solution to the travelling salesperson problem, which forms the basis of all routing problems. As our ultimate goal is to aid urban transport planning, which primarily relies on ground vehicles, we designed the game to encourage users to think in terms of the city-block (Manhattan) distance. To capture the instinctive decision-making process of the users, we score both on the shortness of the path constructed and the time taken to solve the problem. To the best of our knowledge, PathGame is the only solution in existence, with these features.

We have also used the initial deployment of our game to collect over 16,000 tours, containing more than 22 million destinations. The data, which are freely available for research purposes, are the largest dataset to date, dealing with the non-Euclidean TSP and are collected for problems larger than those typically used in previous studies. This allowed the problem-solving behaviour to be anlayzed not previously captured and show that humans, when instructed to provide a solution as quickly as possible, actually seem to do solve the TSP in practically constant time regardless of the problem complexity and that, when the density of destination points increases above a certain point, they solve the problem by simply following a geometric pattern. The human solutions limited to those not following the geometric strategy, including those beating computer-generated solutions based on nearest neighbor and simulated annealing heuristics, actually take time that depends logarithmically on the complexity of the problems. These effects have not been reported on before.

## Figures and Tables

**Table 1:** Summary of models for the relationship of time and problem complexity.

Model summary			Parameters and fitness criteria				
No.	Complexity	Functional form	RMSE	SSE	*a*	*b*	*c*
1	*O*(ln *n*)	*y*=*a* ln(*x*+*c*)+*b*	**11.6571**	**1.1065 × 10** ^**6**^	5.092	0.7428	−4.436
2	*O*(*n*)	*y*=*ax*+*b*	11.7223	1.1191 × 10^6^	0.07247	15.67	
3	*O*(*n* ln *n*)	*y*=*a* ln(*x*+*c*)+*b*	11.7232	1.1191 × 10^6^	0.007796	−237.3	3922
4	*O*(*n*^2^)	*y*=*a*(*x*+*c*)^2^+*b*	11.7286	1.1201 × 10^6^	2.844 × 10^−5^	−23.45	1176
5	*O*(*n*^3^ ln *n*)	*y*=*a*(*x*+*c*)^3^ln(*x*+*c*)+*b*	11.7673	1.121 × 10^6^	2.889 × 10^−8^	13.3	275.5
6	*O*(*n*^2^ ln *n*)	*y*=*a*(*x*+*c*)^2^ln(*x*+*c*)+*b*	11.7332	1.1276 × 10^6^	6.339 × 10^−6^	−4.276	699.5
7	*O*(*n*^3^)	*y*=*a*(*x*+*c*)^3^+*b*	11.7473	1.1237 × 10^6^	6.791 × 10^−8^	7.856	501.6

## Data Availability

The online game is deployed to https://pathgamefts.herokuapp.com and can still be played. The data used in this study and the source code of our game have been deposited on GitHub (http://dculibrk.github.io/pathgame) and made freely available for research purposes.
